# Evaluation of Microstrain in the Regions Surrounding Morse Taper and External Hexagon Implants

**DOI:** 10.1055/s-0044-1787696

**Published:** 2024-07-16

**Authors:** André Luiz de Melo Moreno, Marcio Campaner, Clóvis Lamartine de Moraes Melo Neto, Nathaly Vilene de Araújo Moreno, Daniela Micheline dos Santos, Marcelo Coelho Goiato

**Affiliations:** 1 Department of Dental Materials and Prosthodontics, São Paulo State University (UNESP), School of Dentistry, Araçatuba, São Paulo, Brazil; 2 Courses in Dentistry (IEEP - Instituto de Excelência em Ensino e Pesquisa), Manaus, Amazonas, Brazil

**Keywords:** strain gauge measurements, dental implant, external hexagon, morse taper, Ti-Base abutment, microstrain

## Abstract

**Objective** The aim of this study was to compare the Morse taper (MT) + titanium base (Ti-Base) abutment with the external hexagon (EH) + Ti-Base abutment by using the strain gauge method in the mesial, distal, and apical–buccal areas around these types of implants.

**Materials and Methods** This study investigated two groups, MT and EH, each comprising five polyurethane samples with a dental implant (3.75 × 11.5 mm) in the area of artificial tooth 15. The strain gauges were glued to the mesial, distal, and apical–buccal polyurethane areas of all samples in relation to the implant. Ti-Base nonangled abutments were installed on the implants in each group. Ten identical zirconia crowns were constructed by scanning and milling and were subsequently cemented onto the Ti-base abutments with calcium hydroxide cement. Then, an axial load of 100 N was applied to the occlusal region of the zirconia crowns, and strain gauge measurements were taken.

**Statistical Analysis** Strain gauge data were assessed by a two-way analysis of variance (ANOVA) with “implant connection” and “strain gauge position” factors, followed by the Bonferroni test ( *p* < 0.05).

**Results** The MT group showed significantly lower microstrain values in the mesial and apical strain gauges compared to the EH group.

**Conclusion** The MT group exhibited less microstrain in the mesial and apical areas of the polyurethane samples near the implant. Consequently, the MT connection was considered more biomechanically advantageous.

## Introduction

Dental implants and prostheses on implants are the gold standard treatment for partially or totally edentulous patients. 1 2 This type of treatment has grown in popularity because, unlike other treatment options, it preserves the adjacent tooth and bone structures. 3 In terms of revenue, the global dental implants and prosthetics market was estimated to be worth $10.4 billion in 2023, and is expected to rise in value to $16 billion by 2029, representing a 7.5% compound annual growth rate over this period. 4 This market growth is directly related to the global aging population, as the risk of tooth loss increases with age. 1 3

Morse taper (MT) and external hexagon (EH) connections are widely used in dentistry. 2 5 MT is an internal connection at a conic angle of 8, 11, or 16 degrees. 5 This connection is created by “a cone within a cone,” which generates a mechanical lock and is strengthened by a screw retention system. 5 Following implant placement, the MT platform is often located 1 to 2 mm below the bone crest, 6 which can improve gingival conditioning and aesthetics of the region. 7 8 In contrast, the EH connection is external, with the abutment fitting over the 0.7 mm-high hexagon of the implant, and the fixation between them is exclusively through screwing. 5 After implant installation, the EH platform is often located at the bone crest level. 8 9 It is worth noting that the MT connection leaves a space between the alveolar bone and the implant–abutment union, whereas the EH connection does not. Furthermore, MT results in a more effective seal than EH. 2 5 10

In the field of oral rehabilitation, digital workflow reduces work time and creates high-precision prosthetic restorations. 11 This method associates intraoral scanning with the computer-aided design/computer-aided manufacturing (CAD/CAM) system. 11 CAD/CAM-compatible titanium base (Ti-Base) abutments have been used as a platform to mill customized ceramic prostheses. 11 12 After being milled and sintered, the restoration is cemented to the Ti-Base abutment outside the mouth, which is then screwed to the dental implant (hybrid-abutment prosthesis). 11 12 The advantages of this hybrid-abutment prosthesis concept include aesthetics, the absence of ceramic material at the level of the implant–abutment connection, and a cementation procedure under controlled environmental conditions (outside the mouth), which also facilitates the removal of excess cement (improved biomechanical performance). 11 12

The strain gauge method is used *in vitro* to evaluate the microdeformation of structures. 12 According to Goiato et al 13 :

“Strain gauges are small electrical resistances that, on undergoing minimal deformation, alter the resistance created to the low-intensity current that runs through them and measure the deformation undergone by the object to which they are applied. The electrical signal captured is sent to a data acquisition board to be transformed into a digital signal, enabling it to be read on a computer. These small extensometry terminals have the capacity to record, with great precision, any deformation that occurs when they are submitted to the action of a stress. Strain gauges may be used to assess stresses in prostheses, implants, and teeth.”

A PubMed search combining the keywords “extensometry” and “Ti-Base” or “strain gauge” and “Ti-Base” or “extensometry” and “Morse taper” and “external hexagon” or “strain gauge” and “Morse taper” and “external hexagon” did not reveal articles comparing MT/Ti-Base with EH/Ti-Base using the strain gauge measurement method. Thus, the objective of this study was to compare the MT + Ti-Base abutment with the EH + Ti-Base abutment by using the strain gauge method in the mesial, distal, and apical–buccal areas around these types of implants.

## Materials and Methods

### Groups

The study consisted of two groups, each with a different connection system, and included five polyurethane samples (F160 AXSON, Brazil) 2 :

- EH group: Each polyurethane sample consisted of one EH implant (HE TI BIOFIT, DSP, Brazil) along with one nonangled Ti-Base abutment measuring 5.0 × 4.7 mm (base height: 1.0 mm) (Ti-Base Standard HE, DSP, Brazil) and a single zirconia crown.
- MT group: Each polyurethane sample consisted of one MT implant (CMi, DSP, Brazil) along with one non-angled Ti-base abutment measuring 5.0 × 4.7 mm (base height: 1.5 mm) (Ti-Base Standard CMi, DSP, Brazil) and a single zirconia crown.

The implants in both groups shared identical dimensions (3.75 × 11.5 mm), external design, and surface treatment, and were manufactured with the same type of metal.

### Sample Manufacturing

We used a dental manikin (PD100 Top Dentística, Pronew, Brazil) missing artificial tooth 15 to manufacture 10 polyurethane samples (F160 AXSON, Brazil).

**Fig. 1:**
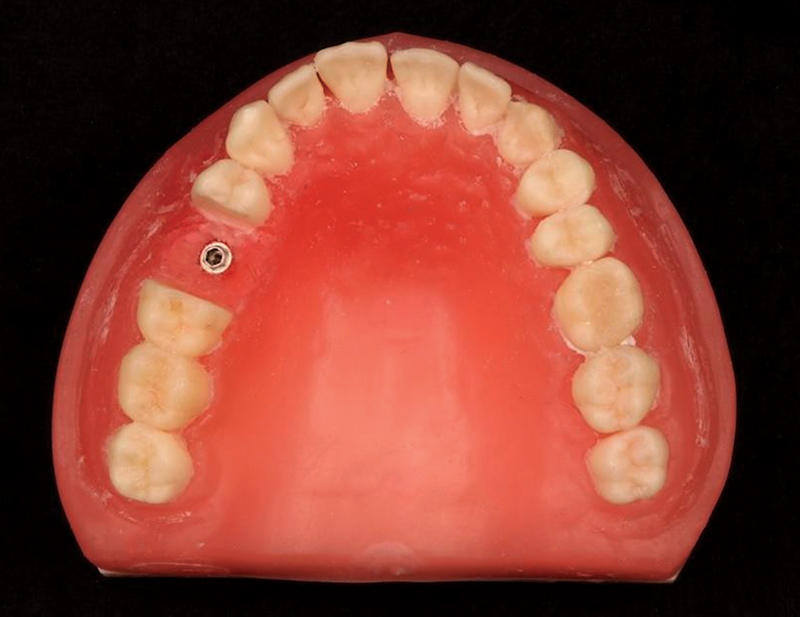
Dental manikin with an external hexagon (EH) implant fixed in the region of artificial tooth 15.

**Fig. 2:**
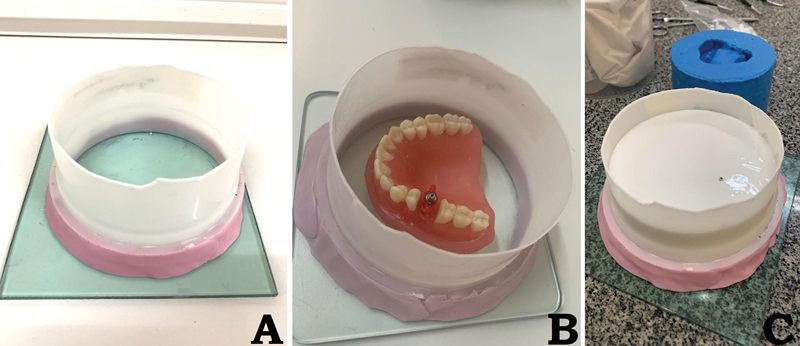
Sample creation sequence. ( **A** ) A polyvinyl chloride (PVC) tube fixed with Zetalabor (Zhermack, Italy) on a glass plate. ( **B** ) The manikin positioned in the center of the PVC tube with the transfer screwed to the implant. ( **C** ) Handcrafted silicone inside the PVC tube until almost covering the transfer.

Initially, with the aid of a dental surveyor, the EH implant was positioned in the edentulous area of the dental manikin and later fixed with a self-polymerizing acrylic resin (Pattern Resin LS, GC, United States). To position the implant in the edentulous area, it was first screwed to the screw of its open tray transfer (DSP, Brazil); then, the transfer screw was screwed to the dental surveyor; and, finally, the implant was positioned in the edentulous area using the dental surveyor. The use of a surveyor was necessary to standardize the position of inclusion of each type of implant in the manikin, which was slightly mesialized to reflect the natural tooth inclination (second premolar of the maxilla). 14 The inclination of the implant was similar to that of the first premolar of the maxillary manikin. After positioning the EH implant in the edentulous area, acrylic resin increments were placed around the implant to fix it to the manikin. The screw of the open tray transfer (DSP, Brazil) was released from the surveyor following acrylic resin polymerization. A wax layer (Wilson, Brazil) was applied to the acrylic resin to obtain the final finish on the edentulous surface ( Fig. 1 ). The EH implant platform was placed at the wax level. Then, transfer molding was performed using silicone (SIQMOL, SIQUIPLÁS, Brazil) ( Fig. 2 ). After silicone polymerization, the mold was separated from the manikin by unscrewing the open tray transfer. A new EH implant (HE TI BIOFIT, DSP, Brazil) was screwed onto the transfer retained in the silicone mold. Subsequently, the F160 polyurethane resin (F160 AXSON, Brazil) 2 15 was manipulated and poured into a silicone mold according to the manufacturer's recommendations. The polyurethane resin was polymerized, then the transfer was removed and the sample was detached from the silicone mold. All the polyurethane samples from the EH group were manufactured using the same mold. The same procedure was repeated for the MT group, but the MT platform was positioned and fixed 1 mm below the wax level.

**Fig. 3:**
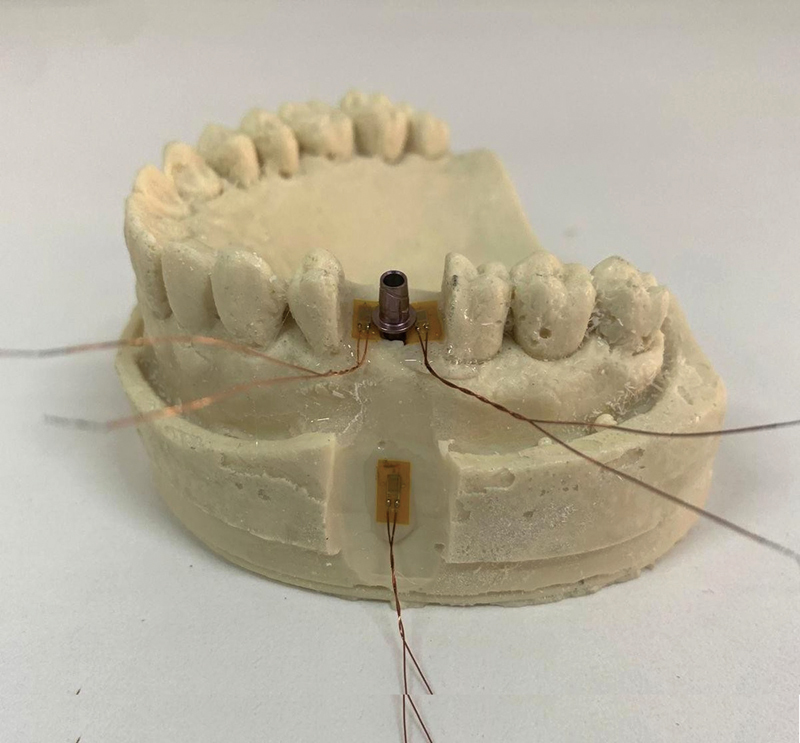
An example sample with strain gauges positioned in the mesial, distal, and apical regions.

The mesial, distal, and apical–buccal polyurethane areas relative to the implant were cleaned with absolute alcohol and dried. Strain gauges (PA06060BA, Excel Sensors Ind. Com. Exp. Ltd., Brazil) already tested on a voltmeter (350 µV) were glued onto these areas with cyanoacrylate ( Fig. 3 ). After drying the cyanoacrylate, the strain gauges were tested again using a voltmeter at the same voltage. The strain gauges were then isolated with a hot-melt adhesive (TRAMONTINA, Brazil) using a hot glue gun ( Fig. 3 ).

### Manufacture of the Single-Unit Crowns

**Fig. 4:**
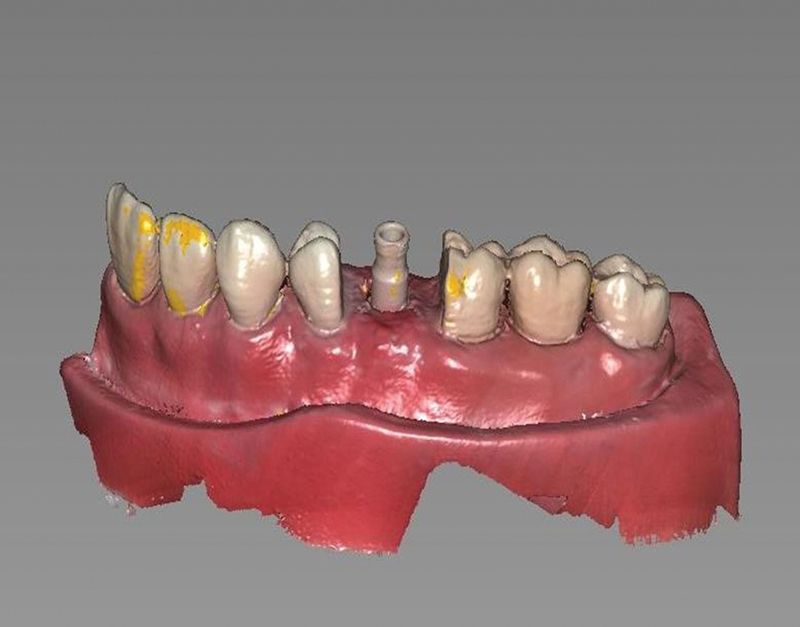
Scanbody DSP scanning procedure.

A scan body (DSP, Brazil) was screwed onto the EH implant placed in the manikin and scanned (SC3600 Scanner, Carestream, United States; Fig. 4 ). The scanned images were used by the dental laboratory to plan the full zirconia crown for cementation onto a Ti-Base abutment. Planning was performed using EXOCAD software (Exocad, Germany). The Ti-Base abutment used for both the EH and MT implants had the same external design and dimensions, meaning a single design was used to mill 10 identical zirconia crowns (Protmat, Brazil). Thus, a crown-manufacturing project created for the EH group was used for the MT group. Next, InlabCAM software (Dentsply Sirona, United States) and an MCX5 milling machine (Dentsply Sirona, United States) were used. After milling, the zirconia crowns were sintered in an oven (InLab Profire Sirona). The crown was made with slight mesialization, similar to that of the first premolar of the maxillary manikin. 14

### Ti-Base Abutment Installation and Zirconia Crown Cementation

**Fig. 5:**
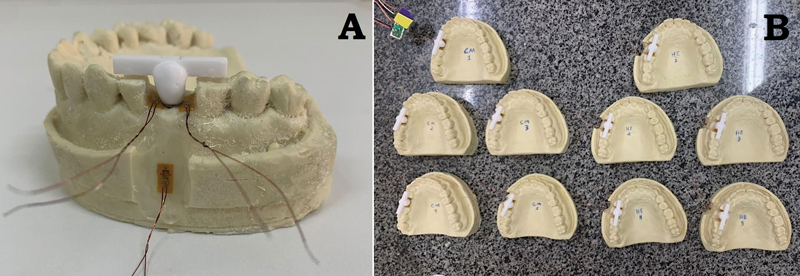
( **A** ) Zirconia crown cemented to the titanium base (Ti-Base) abutment. The zirconia horizontal bar was created to assist in applying axial force. ( **B** ) Finalized groups.

The Ti-Base abutments were screwed to their respective implants in the polyurethane samples using the 30 Ncm torque recommended by the manufacturer. The Ti-Base abutments and the interior part of the zirconia crowns were subsequently cleaned with 70% alcohol and dried. Zirconia crowns were cemented to the abutments with calcium hydroxide cement (Hydro C, Dentsply Sirona, United States), following the manufacturer's recommendations. Immediately after cementation, a load of 5 kg was applied to the crowns for 5 minutes. Cement hardening occurred when this load held the crowns in position ( Fig. 5 ).

### Strain Gauge Measurements

**Fig. 6:**
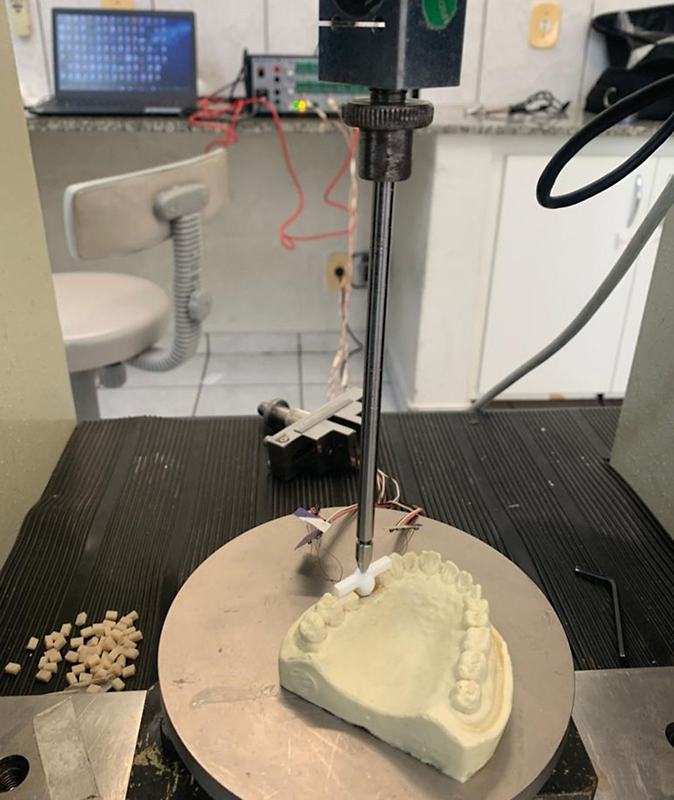
Application of a 100 N axial load and strain gauge measurement.

A 100 N axial load was applied to the center of the occlusal surface of each sample using a universal testing machine (EMIC DL-3000, Brazil; Fig. 6 ). 2 The strain gauges were conﬁgured into a one-quarter Wheatstone bridge and the data were transferred through a data acquisition system (ADS2000, Lynx Tecnologia Eletrônica Ltd., Brazil) and processed by specific software (AqDados 7, Lynx, Brazil). 2 Strain was tested five times on each sample to obtain a mean value in microstrain. The tests were conducted when the microstrain values were zero, indicating the absence of plastic strain. 2

### Statistical Analysis

All statistical analyses were performed using the Jamovi software (version 2.2.5.0; Jamovi Project, Australia). The Shapiro–Wilk test was used to assess data normality. Strain gauge data were assessed by a two-way analysis of variance (ANOVA) with “implant connection” and “strain gauge position” factors, followed by the Bonferroni test ( *p* < 0.05).

## Results

**Graph 1:**
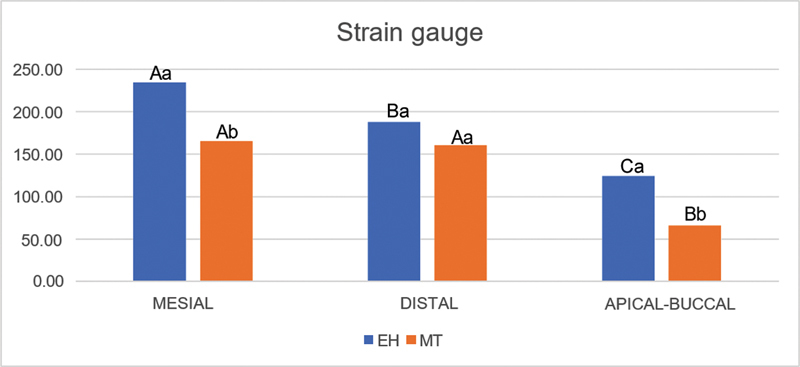
Mean microstrain values for the strain gauge positions of each implant connection. (Bonferroni's test, *p* < 0.05). EH, external hexagon; MT, Morse taper. Different capital letters for the same group (EH or MT) represent a statistically significant difference. Different lowercase letters for the same strain gauge position (mesial, distal, or apical–buccal) represent a statistically significant difference between the MT and EH groups.

**Graph 2:**
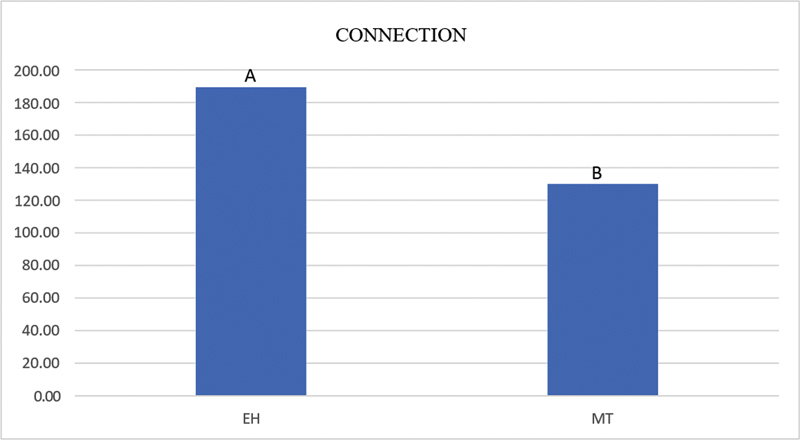
Mean microstrain values for Morse taper (MT) and external hexagon (EH) connections, regardless of the strain gauge position. (Bonferroni's test, *p* < 0.05). Different capital letters represent a statistically significant difference.

All factors evaluated were statistically significant according to the two-way ANOVA test ( Table 1 ). Graph 1 and Table 2 show that the EH group exhibited significantly higher microstrain values than the MT group at the mesial and apical positions. In the EH group, the mesial strain gauge showed a significantly higher microstrain value than those in the other positions, and the distal strain gauge showed a significantly higher microstrain value than that in the apical position. In the MT group, the mesial or distal strain gauges showed a significantly higher microstrain value than that in the apical position. As shown in Graph 2 , the EH group exhibited a significantly higher microstrain than the MT group based only on the type of connection.

**Table 1:** Analysis of variance (ANOVA)

**Source**	**Type III sum of squares**	**df**	**Mean square**	***F***	**Significance**
Corrected model	127,927.251a	9	14,214.139	284.261	0.000 a
Intercept	1,272,953.724	1	1,272,953.724	25,457.103	0.000 a
Position	54,079.392	4	13,519.848	270.376	0.000 a
Connection	44,065.898	1	44,065.898	881.250	0.000 a
Position × connection	29,781.961	4	7,445.490	148.898	0.000 a
Error	2,000.155	40	50.004		
Total	1,402,881.130	50			

a Statistical significance ( *p* < 0.05).

**Table 2:** Mean microstrain values and standard deviation for the strain gauge positions of each implant connection

	**EH**	MT
Mesial strain gauge	235 ± 18.7 A,a	165.5 ± 37.2 A,b
Distal strain gauge	188 ± 52.4 B,a	160 ± 9.7 A,a
Apical strain gauge	124.4 ± 80 C,a	65.8 ± 34.9 B,b

Abbreviations: EH, external hexagon; MT, Morse taper.

Note: Bonferroni's test, *p* < 0.05. Different capital letters in the vertical columns for the same group (EH or MT) represent a statistically significant difference. Different lowercase letters in the horizontal rows for the same strain gauge position (mesial, distal, or apical–buccal) represent a statistically significant difference.

In both groups, there were no mechanical complications after applying a force of 100 N to the samples, such as screw loosening or fractures.

## Discussion

In this study, the mean mesial and distal microstrain values were significantly higher than the apical values in both the EH and MT groups. This may have occurred because the axial force applied to the occlusal region of the crowns was closer to the implant platforms than to their apices ( Table 2 and Graph 1 ). Notably, the apical microstrain was significantly lower in the MT group than in the EH group. This suggests that the MT connection more effectively dissipated stress toward the implant apex, resulting in a significantly lower level of microstrain in the apical-buccal polyurethane area.

Okeson stated that the natural maxillary premolars are slightly mesially inclined. 14 In the present study, implants and crowns followed this principle and, when associated with the characteristics of the EH connection, 2 5 this may explain the significantly higher levels of mesial microstrain compared to distal microstrain in the EH group ( Table 2 and Graph 1 ). It is possible to consider this explanation when observing the absence of significant difference between the values of the mesial and distal strain gauges in the MT group. It is also noteworthy that the mesial microstrain in the MT group was significantly lower than that in the EH group. This suggests that the MT connection likely dissipated more stress than the EH connection, leading to a significantly lower level of microstrain in the mesial polyurethane area. This indicated that this internal connection was not influenced by a slight mesial inclination of the implant and crown. The characteristics of the MT connections may explain this result. 2 5

From a biomechanical standpoint, a significantly lower microstrain value around the implant platform may suggest less bone resorption in this region over time, 2 16 which is crucial for ensuring implant stability and survival, particularly for short implants. 16 Additionally, bone maintenance plays a vital role in maintaining aesthetics. The positioning of the implant 1 mm below the polyurethane crest may also have decreased the mesial microstrain in the MT group. However, a study comparing microstrain at different depths of MT implant installations is needed to confirm this hypothesis.

The primary limitation of this study is the absence of a comparison between different abutments. Furthermore, future studies need to be conducted to test the samples using oblique forces.

## Conclusion

The MT group exhibited less microstrain in the mesial and apical areas of the polyurethane samples close to the implant. Consequently, the MT connection was considered more biomechanically advantageous.
